# Pliocene sigmodontine rodents (Mammalia: Cricetidae) in northernmost South America: test of biogeographic hypotheses and revised evolutionary scenarios

**DOI:** 10.1098/rsos.221417

**Published:** 2023-08-02

**Authors:** Christophe Ronez, Jorge D. Carrillo-Briceño, Patrícia Hadler, Marcelo R. Sánchez-Villagra, Ulyses F. J. Pardiñas

**Affiliations:** ^1^ Instituto de Diversidad y Evolución Austral (IDEAus-CONICET), Boulevard Brown 2915, 9120 Puerto, Madryn, Argentina; ^2^ Department of Paleontology, University of Zurich, Karl-Schmid-Straße 4, 8006 Zurich, Switzerland; ^3^ Universidade Federal de Santa Catarina, João David Ferreira Lima, s/n, Florianópolis, Brazil; ^4^ Associate Researcher, Instituto Nacional de Biodiversidad (INABIO), Quito 170135, Ecuador

**Keywords:** biogeography, *Oligoryzomys*, Oryzomyini, San Gregorio formation, Sigmodontinae, *Zygodontomys*

## Abstract

We document the first occurrence of Sigmodontinae (Mammalia, Rodentia, Cricetidae) from the Pliocene of northern South America, from the San Gregorio Formation of northwestern Venezuela. The recovered isolated molars are identified as *Oligoryzomys* sp. and *Zygodontomys* sp., two currently widespread sigmodontines in South America. These records constitute the oldest representatives of these genera, potentially new species, and the first Pliocene occurrence for Oryzomyini and the whole subfamily outside Argentina. Hypotheses on the historical biogeography of sigmodontines have been constructed almost exclusively using genetic data and the fossils we report provide a new kind of evidence. The occurrence of *Oligoryzomys* sp. and *Zygodontomys* sp. in Venezuela provides novel information for the diversification models suggested for *Oligoryzomys*, by supporting a potential eastern corridor of open environments from northern to southern South America. The presence of sigmodontines from the locality home of the new reports, Norte Casa Chiguaje, is consistent with the palaeoenvironmental conditions originally proposed for it based on mammals and botanical records, being characterized as mixed open grassland/forest areas surrounding permanent freshwater systems. The new sigmodontine evidence is used to discuss the putative scenarios of the ancient evolution of the subfamily in South America, favouring a model in which open areas (savannahs) to the east of the Andes played crucial role aiding or obstructing Late Miocene–Pliocene sigmodontine dispersion southwards.

## 1. Introduction

South America harbours most of the living diversity of the Sigmodontinae, the richest group of cricetid rodents. Recent accounts indicate the clade is comprised at least 95 extant genera, including those that became extinct during historical time [1]. Most of them (except †*Cordimus*, †*Megalomys*, †*Pennatomys* and *Rheomys*) are recorded between the Atrato River (Colombia) and the Cape Horn Islands in the southernmost part of the continent [2–4]. Due to the great diversity in modern genera, it has been thought that South America was the location of most of the group's evolutionary history [5–9]. However, this is undermined by the scarcity of palaeontological evidence [1,10,11].

The oldest non-controversial evidence on fossil sigmodontines comes from central Argentina [12]. Although the age of the fossil-bearing deposits has been disputed [1,13], they seem to be from the latest Miocene according to recent refined faunal analyses and new absolute dates [14]. The palaeontological record of sigmodontines outside Argentina is restricted to younger deposits, typically not older than Late Pleistocene [11,15] or controversial Middle–Late Pleistocene (e.g. Tarija basin [16–18]). Therefore, a sharp asymmetry characterizes the Sigmodontinae fossil history, with a long and crucial segment biased to high latitudes and little and late evidence from currently tropical and subtropical huge regions [19–22].

**Figure 1.**
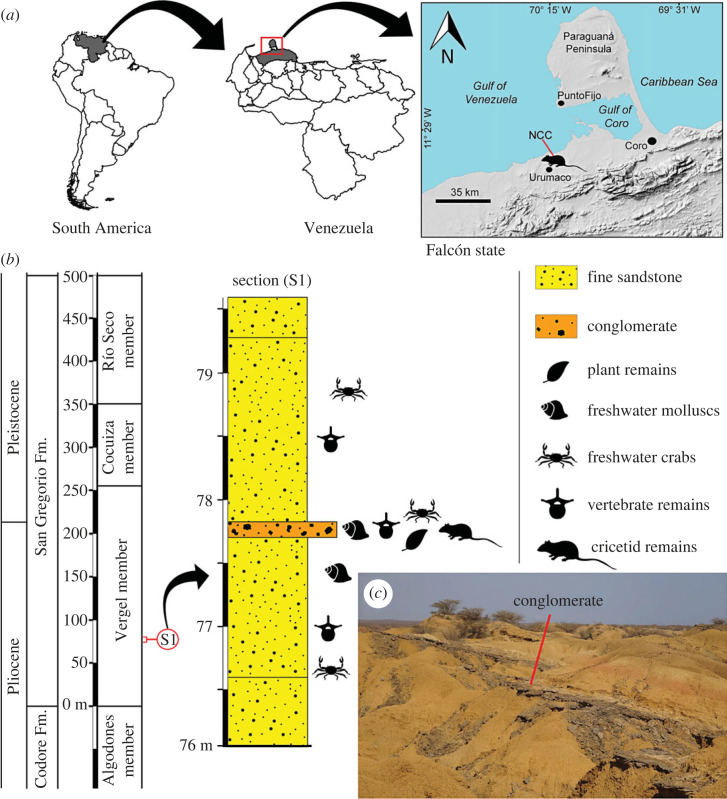
Localization and stratigraphic record of the Norte Casa Chiguaje locality (Pliocene, Venezuela). (*a*) Geographical location of the studied locality (11°17′52.9″ N, 70°14′7.3″ W). (*b*) Generalized section of Norte Casa Chiguaje locality of the Vergel Member, San Gregorio Formation (based on Carrillo-Briceño *et al.* [23]). (*c*) Photograph showing a general view of the fossiliferous outcrops at NCC (from [23, fig. 2*a*]).

In this contribution we describe the first Pliocene sigmodontines recovered in South America outside Argentina. Paradoxically, they come from the northernmost portion of the continent, near the Caribbean coast of western Venezuela, and are part of a rich fossil vertebrate assemblage recently documented [23] (figure 1). Main biogeographical and systematic aspects derived from this noteworthy finding are addressed, including testing of models advanced to explain the historical biogeography of the subfamily [24–29].

## 2. Geological setting

All specimens came from the locality of Norte Casa Chiguaje locality (NCC; 11°17′52.9″ N, 70°14′7.3″ W) of the Vergel Member, San Gregorio Formation (Fm.), Falcón State, Venezuela (figure 1). The San Gregorio Fm. is the youngest unit of the Urumaco stratigraphic sequence [30], with an estimated thickness of 570 m at it type section [31–33]. The San Gregorio Fm. is divided into three formal members [31]: Vergel (lower), Cocuiza (middle), and Río Seco (upper). The Vergel Member is an approximately 260 m-thick sequence [33] composed of interbedding mudstones, sandstones and sparse conglomeratic beds that denote fluvial environments (e.g. braided rivers) and alluvial fans [30–33]. The area where the Vergel Member outcrops is currently characterized by arid conditions and badlands, offering excellent exposures of the fossil-bearing layers (figure 1*c*; see fig. 2*a–f* in Carrillo-Briceño *et al*. [23]).

The NCC locality and section (figure 1*b*) is characterized by unconsolidated yellowish-orange to light-brown fine sandstones with a thin (approx. 30 cm) consolidated conglomeratic layer of a light–dark grey matrix with well-rounded to sub-rounded clasts of up to 25 mm in diameter (figure 1*c*; see fig. 2*a–f* in Carrillo-Briceño *et al*. [23]). From this single locality, abundant macro and micro fossil remains of at least 49 continental taxa of stingrays, bony fishes, amphibians, crocodiles, lizards, snakes, aquatic and terrestrial turtles and mammals were reported [23]. The vertebrates and palaeobotanical elements found in NCC suggest mixed open grassland/forest areas surrounding permanent freshwater systems [23].

In reference to the antiquity of the Vergel Member, a Late Pliocene age has been proposed based on its stratigraphical position, palynological content, and the associated mammalian assemblage [23,34–36]. It is also supported by the Early Pleistocene age of the overlying Cocuiza Member based on its nannoplankton content and the ^86^Sr/^88^Sr dating (figs 3 and 30 in Carrillo-Briceño *et al*. [23]).

## 3. Material and methods

The cricetid sample from the NCC locality consists of eleven specimens (five complete molars, three partial molars, one upper incisor and two lower incisors) identified in this study as belonging to three different taxa. The results presented here apply to two of these taxa because the third taxon is still under study. Also, the systematic status of certain specimens cannot be determined (electronic supplementary material, figure S1). The specimens were recovered through screen-washing (conducted by J.D.C.-B.), using standard sieves with up to 0.5 mm mesh, of a total of approximately 250 kg of conglomerates and unconsolidated fine sandstones from the NCC locality of the Vergel Member (San Gregorio Fm.). All specimens are housed in the Colección Paleontológica de la Alcaldía Bolivariana de Urumaco, Falcón State, Venezuela. Dental nomenclature follows Reig [37] and Barbière *et al*. [38] when possible (see electronic supplementary material, figure S2). Taxonomic identifications were achieved via comparison with recent reference specimens (electronic supplementary material, S1) and literature [39–44]. Measurements are in millimetres and correspond to maximum length and width; photographs illustrating studied specimens were obtained employing a scanning electronic microscope (JEOL JSM-6010) in the University of Zurich.

Institutional abbreviations: AMNH, American Museum of Natural History, New York, USA; AMU-CURS, Colección Paleontológica de la Alcaldía Bolivariana de Urumaco, Falcón State, Venezuela; CNP, Colección de Mamíferos del Centro Nacional Patagónico, Puerto Madryn, Argentina; FMNH, Field Museum of Natural History, Chicago, USA; LACM, Los Angeles County Museum of Natural History, Los Angeles, USA; MACN, Colección Nacional de Mastozoología, Museo Argentino de Ciencias Naturales ‘Bernardino Rivadavia’, Buenos Aires, Argentina; MVZ, Museum of Vertebrate Zoology, Berkeley, USA; ZFMK MAM, Collection of Mammalogy of the Zoological Research Museum Alexander Koenig, Bonn, Germany.

Anatomical abbreviations: M1, M2, M3, m1, m2, m3, upper (M) and lower (m) first, second, and third molars, respectively.

## 4. Systematic palaeontology

### 4.1. *Zygodontomys* sp.

Mammalia Linnaeus, 1758

Rodentia Bowdich, 1821

Cricetidae Fischer, 1817

Sigmodontinae Wagner, 1843

Oryzomyini Vorontsov, 1959

Genus *Zygodontomys* Coues, 1874

*Zygodontomys* sp.

**Figure 2.**
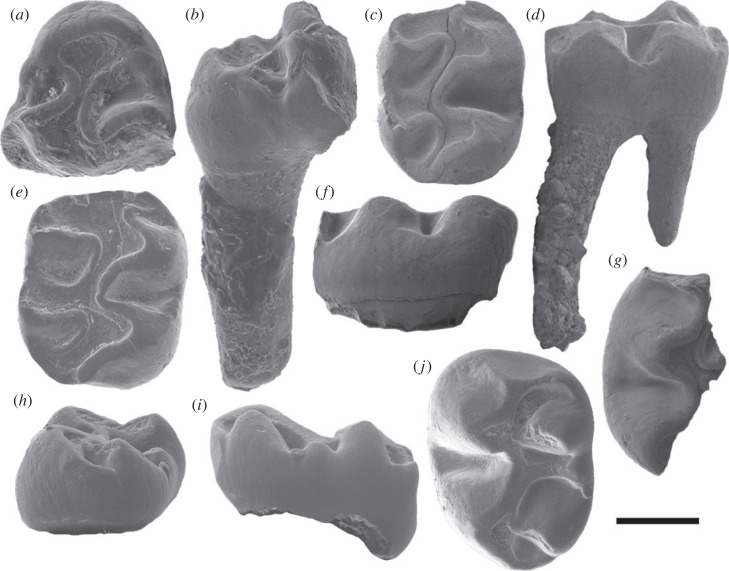
SEM photographs of the sigmodontine molars collected at the Norte Casa Chiguaje locality (Venezuela). (*a–e*) *Zygodontomys* sp. (*a,b*) Anterior portion of a left M1 (AMU-CURS-854); (*c,d*) right m2 (AMU-CURS-1208); (*e*) right m2 (AMU-CURS-853). (*f–j*) *Oligoryzomys* sp. (*f,g*) Lingual portion of a left M1 (AMU-CURS-1209); (*h–j*) left M2 (AMU-CURS-856). Views: labial (*b,d,i*) lingual (*f*), occlusal (*a,c,e,g,j*) and posterior (*h*). Scale bar represents 0.5 mm.

Figure 2*a–e*. Material. AMU-CURS-853: right m2; AMU-CURS-854: anterior portion of left M1; AMU-CURS-1208: right m2. Measurements are provided in table 1.

**Table 1.** Measurements (in mm) of the molars recorded at Norte Casa Chiguaje (Pliocene, Venezuela).

taxa	specimen	length	width
*Oligoryzomys* sp.	AMU-CURS-856 (M2)	1.26	1.05
*Zygodontomys* sp.	AMU-CURS-853 (m2)	1.00	1.30
*Zygodontomys* sp.	AMU-CURS-1208 (m2)	0.85	1.00

#### 4.1.1. Description

AMU-CURS-854 is restricted to the procingulum plus the anterior faces of the protocone and paracone, and belongs to an apparently old individual based on the observed wear (figure 2*a*,*b*). The procingulum is relatively wide, with a connection to the protocone placed centro-lingualy to the medial axis. The protoflexus is shorter than the paraflexus, the latter being slightly curved as it rounds the anterolingual base of the paracone. The two m2s (AMU-CURS-853 and 1208) are complete, showing alternating cusps and a two-rooted condition (figure 2*c–e*). The labial cusps are larger than the lingual cusps. A small anterolabial cingulum is visible, separated from the protoconid by a small protoflexid. All flexids do not overlap each other at the midline axis. The entoflexid penetrates anteriorly at the level of the protoconid. In AMU-CURS-853, there is a small mesostylid and a small ectostylid (figure 2*e*). The hypoflexid is wider than the other flexids. A posteroflexid is present but relatively small, probably due to the well-developed posterior cingulum.

#### 4.1.2. Comparison

The m2s show a simple occlusal pattern lacking mesolophids or additional accessory structures; the broken M1 also suggests an animal with simplified dentition because at least the anteroloph is absent. In addition, the molars are moderately hypsodont. The combination of both traits, occlusal simplification and hypsodonty, sets apart the NCC locality specimens from many sigmodontine clades (e.g. most of the members of Abrotrichini, Akodontini, Oryzomyini, Thomasomyini) characterized by complex (i.e. pentalophodont or tetralophodont pattern according to Hershkovitz [5]) and brachydont molars.

**Figure 3.**
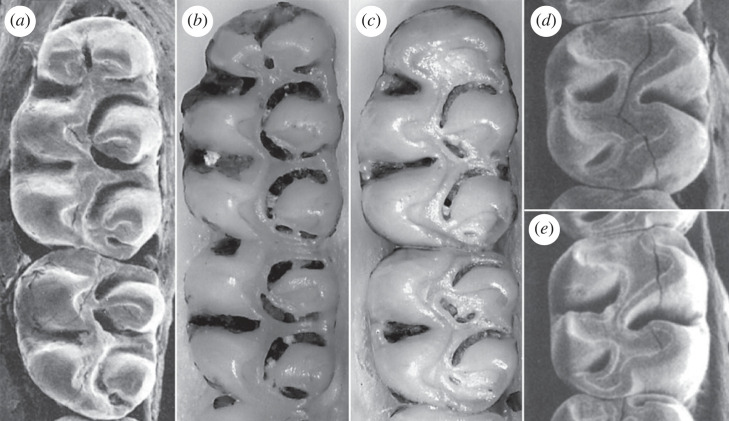
Occlusal view of left first and second upper, and right second lower molars of extant representative of *Oligoryzomys* and *Zygodontomys* used for comparison. (*a*) *Oligoryzomys fulvescens*, AMNH 181446 (left M1 and M2; from Weksler [41]). (*b*) *O. flavescens*, CNP 5536 (left M1 and M2). (*c*) *O*. *nigripes*, CNP 5611 (left M1 and M2). (*d*) *Zygodontomys brunneus*, FMNH 71258 (right m2; from Voss [40]). (*e*) *Z*. *brevicauda*, AMNH 173971 (right m2; from Voss [40]). All scaled to the same size.

Among oryzomyines currently inhabiting northern Venezuela, a reduced posteroflexid in m2 is observable in *Zygodontomys*, a genus that combines a simplified occlusal pattern and hypsodonty (figure 3*d*,*e*). Voss [40, p. 20] specified that the condition of *Zygodontomys* is ‘anterocone(id) of M1/m1 undivided; …; mesolophs and mesolophids entirely absent’. All species of *Zygodontomys* show a connection between the procingulum and the protocone that is situated centrally in young and adult specimens [40]. However, this connection is slightly shifted lingually in older specimens. Table 2 lists selected traits comparing studied fossils and *Zygodontomys* species. Observed similarities are enough to support generic assignment, although one main difference is related to the number of roots (two roots versus three in the extant species, see below).

**Table 2.** Comparison of selected dental traits among *Zygodontomys* sp. from Norte Casa Chiguaje locality (Pliocene, Venezuela), *Zygodontomys brevicauda*, and *Z*. *brunneus*.

character	*Zygodontomys* sp.	*Z. brevicauda*	*Z. brunneus*
procingulum	wide	wide	wide, almost as large as M1
anterior platform	absent	present	absent
dentine in procingulum	rounded (reduced labio-lingualy)	labio-lingualy compressed	labio-lingualy compressed
connection between procingulum and protocone	inclined	straight	inclined
protoflexus	small and continuous	small, anterior shift	small, anterior shift
paraflexus	separates protocone and paracone	smaller, protocone and paracone well in contact	separates protocone and paracone
number of roots on m2	2	3	3
mesostylid	small	small	absent
connection between protoconid and entoconid	not aligned with the protoconid	not aligned with the protoconid	aligned with the protoconid
hypoconid–entoconid alternation	alternate	opposite	slightly alternate
hypoflexid	wide and slightly curved posteriorly	curved posteriorly	curved posteriorly

### 4.2. *Oligoryzomys* sp.

Genus *Oligoryzomys* Bangs, 1900

*Oligoryzomys* sp.

Figure 2*f–j*. Material. AMU-CURS-1209: lingual portion of left M1; AMU-CURS-856: left M2. Measurements are provided in table 1.

#### 4.2.1. Description

AMU-CURS-1209 is identified a partial M1; although restricted to the lingual side of the tooth, several preserved occlusal features are enough to confirm its anatomical position and advance a taxonomical hypothesis (figure 2*f*,*g*). Its anterior part indicates the presence of a procingulum. The protocone shows an anterior arm going antero-labialy to the procingulum, but its lingual side likely implies a connection with the lingual conule. Posteriorly, the protocone connects with the paracone in the posterior portion of its maximum dentine exposure and directly with the hypocone, via both the anterior arm of the hypocone and the hypo-anterolophule, respectively. The protoflexus is slightly curved posteriorly, whereas the hypoflexus is wide and straight. AMU-CURS-856, a left M2 (figure 2*h–j*), has the main cusp pairs (paracone–protocone and metacone–hypocone) opposing and shows a crested occlusal surface [5,45]. The tooth is sub-oval in outline and the light occlusal wear suggests a subadult individual. A thin anteroloph (anterior cingulum) is present as an enamel ridge and closes the paraflexus labially. The protocone connects anteriorly to the anteroloph by its anterior arm, in the posterior portion of its maximum dentine exposure to the anterior arm of the paracone, and posteriorly, with the hypo-anterolophule. The hypocone has a long and oblique anterior arm—showing no dentine exposure—which divides into a short hypo-anterolophule and longer hypo-mesolophule that connects with the projection from a mesostyle (forming a long mesoloph complex), closing off the mesoflexus. A small cuspule also closes off the metaflexus labially. The metacone is connected to the hypocone through the hypo-endolophule and the meta-anterolophule. The anterior arm of the metacone is oriented towards the middle of the maximum dentine exposure. Posteriorly, the hypocone connects with a posterior cingulum through its posterior arm. The weak development of this small posteroloph complex explains such a general outline. Labial and lingual flexi do not overlap each other at the midline. Both the hypoflexus and the mesoflexus are oriented transversely to the antero-posterior axis, whereas the para- and metaflexus are curved posteriorly but never round their respective cusps. The posteroflexus remains enclosed labially, and appears rounded. No information on the roots could be determined.

#### 4.2.2. Comparison

Both the complexity and crown height of the specimens indicate affinities to sigmodontine taxa with brachydont and complex molars (i.e. members of Oryzomyini, Rhagomyini, Thomasomyini, Wiedomyini and few Sigmodontinae incertae sedis). The connection of the paracone to the anterior part of the protocone is not recorded in Rhagomyini, Thomasomyini, Wiedomyini, and *Delomys* but it is present in several Oryzomyini. Within the latter, the combination of direct connections of the paracone and metacone to the maximum dentine exposures of the protocone and hypocone, respectively, is restricted to *Oligoryzomys* (figure 3*a–c*). Therefore, the material of NCC locality can be confidently referred to this widespread oryzomyine genus.

Table 3 lists the differences among several extant species of *Oligoryzomys* and the material of NCC locality (see also electronic supplementary material, figure S3). The resemblance of several dental traits of the NCC fossil molars to those of *Oligoryzomys fulvescens*, a widespread species in northern South America and Central America [46,47], is remarkable. *Oligoryzomys victus*, a Caribbean species that went extinct during historical times [48], can be separated from the NCC fossils by having an incomplete mesoflexus, not being crossed by a para-mesolophule (isolating an enamel atoll in *O*. *victus*).

**Table 3.** Comparison of selected dental traits among *Oligoryzomys* sp. from Norte Casa Chiguaje locality (Pliocene, Venezuela), and a sample of species of the genus.

character	*O.* sp*.*	*O. nigripes*	*O. mattogrossae*	*O. fulvescens*	*O. flavescens*	*O. rupestris*	*O. victus*	*O. moojeni*	*O. longicaudatus*
conection of the paracone on the protocone (M1)	posterior	at the middle	at the middle	posterior	at the middle	at the middle	at the middle	at the middle	posterior
conection of the paracone on the protocone (M2)	at the middle	anterior	anterior	at the middle	anterior	anterior	anterior	anterior	at the middle
connection of the metacone on the hypocone	at the middle	at the middle	?	posterior	anterior	posterior	at the middle	anterior	posterior
anterior enamel atoll (M2)	absent	present	present	present	absent	absent	present	present	present
posterior enamel atoll (M2)	absent	absent	present	present	present	absent	absent	present	absent
anterolingual cingulum (M2)	few developed	few developed	?	few developed	well developed	few developed	well developed	well developed	well developed

## 5. Discussion

### 5.1. Taxonomy of the sigmodontines from Norte Casa Chiguaje

Our study of fossil *Oligoryzomys* shows that specimens display a mosaic of characters seen in several extant species (figure 3), likely indicating they represent a new species. However, a rigorous taxonomic hypothesis cannot be advanced without examining extensive samples of well-defined species of *Oligoryzomys* from northern South America. Recent systematic revisions in the widespread rodent *Oligoryzomys* have been conducted mostly based on molecular evidence (see [49] and references therein). Although 22 extant species are recognized as valid [3,50–52], at least 33 independent lineages are considered as integrating the genus according to molecular-based phylogenies [53–55]. Most of the recent revisionary efforts focused on *Oligoryzomys* lack any attempt to provide differences based on molar morphology, even for those species regarded as new. An example is the contribution by Hurtado & D'Elía [56, table 1], which presented morphological comparisons among *Oligoryzomys destructor destructor*, *O*. *d*. *spodiurus*, *O*. *andinus* and *O*. *flavescens*. Although this significant study surveyed 30 features, including general body, external, and craniodental traits, not a single aspect of molar morphology was included. A similar set of characters, excluding any from dentition, were used when *Oligoryzomys pachecoi* was described and compared against *O*. *destructor*, *O*. *chaparensis* and *O*. *f*. *occidentalis* [43], or when *Oligoryzomys guille* was named and separated from *O*. *andinus* [52]. Thus, no molar information is available from these studies that would aid in the distinction of these species of *Oligoryzomys*. Contrastingly, Palma & Rodriguez-Serrano [51, fig. 8] used dental morphology when describing *Oligoryzomys yatesi*, as Massoia [57] previously did in his refinement of the several Argentinean species. Given the lack of information about molar variation in most of the alpha-taxonomy of *Oligoryzomys*, fossils can hardly be classified at specific level.

**Figure 4.**
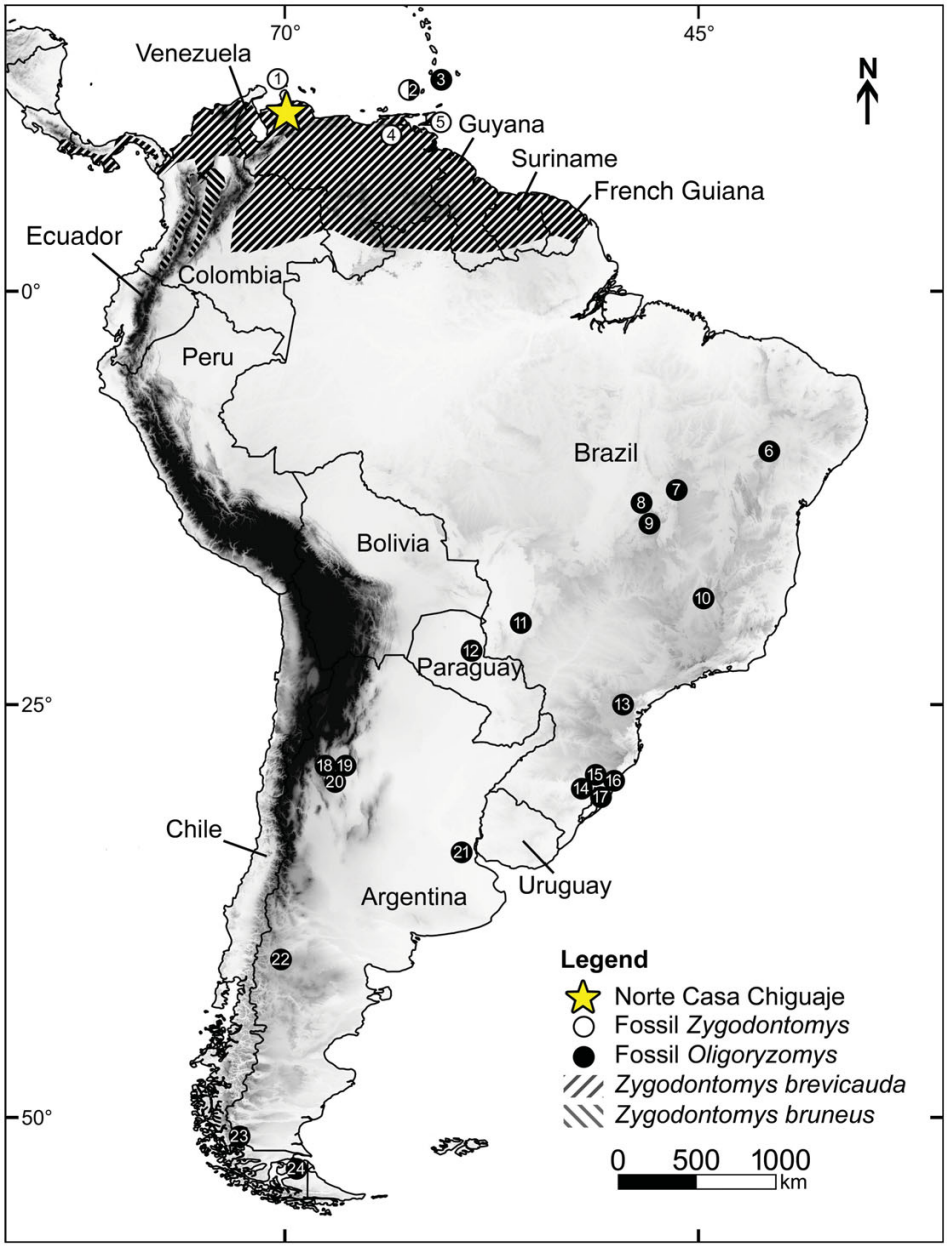
Current geographical distribution of the rodent genus *Zygodontomys* and fossil record attributed to *Oligoryzomys* and *Zygodontomys*: Pliocene record from Casa Norte Chiguaje locality (NCC), and other Quaternary fossil recording localities, including Aruba (1), Cariacou and Grenada (2), Saint Vincent (3), Orocual (4), Trinidad (5), Toca da Boa Vista and Lapa dos Brejões (6), Gruta do Urso (7), Igrejinha (8), Carneiro (9), Lagoa Santa (10), Nossa Senhora Aparecida cave (11), Risso cave (12), Abismo Iguatemi (13), Pilger (14), Sangão (15), Deobaldino Marques (16), Garivaldino (17), La Angostura (18), La Mesada (19), Ruinas Jesuíticas de Lules (20), Cueva Traful 1 (21), Ramallo (22), Cueva del Milodon (23), and Tres Arroyos (24). Constructed from several sources mentioned in the main text.

Hurtado & D'Elía [49] advanced a hypothesis on the biogeography of *Oligoryzomys*. They estimated that the genus first appeared in northern and southern Amazonia, as well as Chaco and the tropical Andes ecoregions, and then spread out to all South and Central American ecoregions. *Oligoryzomys* is considered to have reached the Pacific ecoregion during the Pleistocene (*ca* 1.5 Ma). By contrast, the Venezuelan fossil record firmly indicates that this area was already occupied by a member of the genus by at least the Late Pliocene (around 3.6–2.6 Ma; figure 4). Nonetheless, and taking out the temporality of the events probably due to the lack of calibration (N. Hurtado 2022, personal communication to U.F.J.P.), their phylogeny is in accordance with our morphological comparison. Indeed *O*. *delicatus* and *O*. *fulvescens* share a common ancestor with four other taxa, which correspond to the arrival of the genus to the Pacific ecoregion (fig. 3 in [49]). This fact leads us to propose the NCC taxa as putative calibration to the node 6 of Hurtado & D'Elía [49].

In reference to *Zygodontomys*, it is composed in its current understanding by two extant species: *Z*. *brunneus* and *Z. brevicauda*, the latter including several subspecies [4,58] ranked as full species in some works (e.g. *Z*. *cherriei* [59]). Studies carried out during the last three decades are clearly suggesting that this binary specific taxonomy is unrealistic [58,60–62]. Karyological data revealed different cytotypes allied to *Z*. *brevicauda* [60] while mitochondrial DNA sequences agreed in indicating that the latter is a complex of species [61]. An undescribed new species has been also proposed for an island population along the Pacific coast of Panama, including a complex history for the remainder populations from nearby islands and mainland [59]. A much-needed revision of the entire genus is pending and this situation creates an uncertain alpha-taxonomy scenario to anchor palaeontological findings. At least 10 nominal forms are today subsumed under *Z*. *brevicauda* [58]. Prima facie, the new Venezuelan fossils may represent a new species of the genus, being characterized by greater hypsodonty, more alternate cusp alignment, more planate occlusal surface, and two-rooted condition (figure 3). It is noteworthy that both m2s are two-rooted contrasting with the widespread three-rooted condition in living and Holocene examples of *Zygodontomys*. However, variability in the number of roots is not uncommon in genera with a tendency towards increased hypsodonty (see the discussion about this topic regarding *Sigmodon* in Martin [63]). Alternatively, all these traits could be suggesting that the Venezuelan fossil belongs to a new genus. However, based on the scarcity of the available material we prefer the most parsimonious hypothesis (i.e. to refer it to *Zygodontomys*) which also has the least impact at biological and nomenclatorial level.

Bonvicino *et al*. [61] estimated the age of divergence among members of the *Z*. *brevicauda* clade at 1.9 Ma. Considering that *Zygodontomys* from NCC shows a mosaic of characters shared by *Z*. *brunneus* and *Z*. *brevicauda*, then a putative decrease of diversity within the genus can be surmised. Wing [64, p. 71] stated ‘Although a species of *Zygodontomys* now exists on Trinidad it is not the same species as the fossil form’. Based on karyological and morphological evidence [40,58,61], Trinidadian recent populations belong to *Z*. *brevicauda*; therefore, if Late Pleistocene fossils belong to another species, at least one extinction event and replacement must have occurred. The same is apparently true for the island of Aruba where *Zygodontomys* is recorded in the fossil record, but it is not a part of the extant rodent assemblage [58,65]. Alternatively, this supposed loss of diversity may only be due to the lack of an updated taxonomic revision.

### 5.2. *Oligoryzomys* and *Zygodontomys*: palaeoenvironmental significance

Geographically close to NCC, *Oligoryzomys* has been recorded in Holocene deposits in Granada and Saint Vincent (*O*. *victus* [48,66]). Additional records are far south of the Equator and not older than Middle to Late Pleistocene (figure 4). The oldest remains come from Middle–Late Pleistocene localities in Argentina and are referred to *Oligoryzomys* cf*. O*. *flavescens* [67] and *Oligoryzomys* sp. [68]. Several additional Late Pleistocene occurrences are also known from Argentina (La Angostura and La Mesada localities; *O*. *brendae* and *O*. cf. *O*. *occidentalis* [69]), Brazil (Gruta do Urso, Toca da Boa Vista and Lapa dos Brejões; *O*. cf. *O*. *mattogrossae*, *O*. sp. [70,71]), and Paraguay (Risso cave; *O*. sp. [72]). Holocene localities also yielded *Oligoryzomys* remains from Argentina (La Mesada, Ruinas Jesuíticas de San José de Lules, La Angostura and Cueva Traful 1; *O*. *brendae*, *O*. *longicaudatus*, *O*. cf. *O*. *flavescens*, *O*. cf*. O*. *occidentalis*, *O*. sp. [68,69,73]), Brazil (Lagoa Santa, Gruta do Urso, Pilger, Sangão, Deobaldino Marques and Garivaldino; *O*. *flavescens*, *O*. *nigripes*, *O*. *mattogrossae*, *O*. sp., *O*. cf. *O*. *mattogrossae* [19,21,71,74–78]), and Chile (Cueva del Milodón, Tres Arroyos 1; *O*. *longicaudatus*, *O*. sp. [11,79,80]). Lastly, regarding localities with an indeterminate Quaternary age in Brazil, *O*. cf. *O*. *mattogrossae*, *O*. sp., and cf. *Oligoryzomys* have been reported from Igrejinha and Carneiro, Abismo Iguatemi and Nossa Senhora Aparecida cave, respectively [22,81,82]. The Venezuelan finding described here constitutes the oldest record for the genus and probably represents a new species based on its mosaic of dental characters.

Species of *Oligoryzomys* are currently present from southern North America to southernmost South America, from sea level to around 3500 m in altitude, and are characteristic of very diverse habitats [50]. They occur in lowland and montane forest (Atlantic Forest, lowland Amazonian rainforest and Andean cloud forest), open vegetation such as grassland and shrublands (Cerrados, Pampas, Llanos, Pantanal, Chaco), but also in drier and desertic environments including steppes and tablelands (Patagonia, Caatinga, Peruvian Pacific Coast). For that reason, any attempt of palaeoenvironmental signal based solely on *Oligoryzomys* is pointless. However, the reconstruction as made by Carrillo-Briceño *et al*. [23] is not undermined by the record of *Oligoryzomys* because the palaeoenvironment of NCC has been interpreted to be a mixed forest–grassland based on the fossil assemblage, which includes two niches that the genus inhabits today.

In reference to *Zygodontomys*, to date, only four localities yielded fossil remains of the genus (figure 4). Wing [64] recorded *Zygodontomys* from the Late Pleistocene of Trinidad (assigned to *Z*. *brevicauda* in a posterior revision [83]), and the same was made for cave deposits in the Caribbean island of Aruba [65]. Also regarded as Late Pleistocene, *Z*. *brevicauda* was recovered from El Breal de Orocual (ORS20), a Venezuelan palaeontological asphaltic locality [84]. A Late Holocene sigmodontine assemblage containing numerous *Z*. *brevicauda* from the islands of Carriacou and Granada have been described [44]. Our perception is that these Antillean findings do not represent *Z*. *brevicauda*. Dental differences (e.g. median murid anteroposteriorly oriented and well-defined posterior cusps on m2) are visible between illustrated materials (see figs 7 and 8 in [44]) and typical *Z*. *brevicauda* specimens, but the generic assignment is clearly supported (see also the discussion on p. 18 in Mistretta *et al*. [44]).

Currently, *Z*. *brevicauda* is distributed from southeastern Costa Rica throughout Panama to Colombia, Venezuela, the Guyanas and northern Brazil while *Z*. *brunneus* inhabits the intermountain valleys of Colombia [40,58]. The genus is also recorded, with populations attributed to *Z*. *brevicauda* or *Zygodontomys* sp., in the Lesser Antilles [85]. The fossil occurrence of *Zygodontomys* sp. at NCC sharing traits of both extant species (table 2) likely indicates a more diverse generic past history. As suggested by Carrillo-Briceño *et al*. [23], the Plio-Pleistocene is marked in this area by the establishment of the present climatic condition, that is, one characterized by reduced precipitation and an open vegetation environment [86–89]. This could have been the major factor driving the regional diversification of *Zygodontomys* [61].

*Zygodontomys* is a typical faunistic element characterizing natural or anthropogenic unforested lowland territories (below 1300 m [40]; figure 4). This ground-dwelling tropical sigmodontine is found abundantly in a variety of terrestrial habitats including grasslands, savannah, clearings, marshy areas, secondarily shrubby areas as well as agricultural fields [90,91]. The occurrence of *Zygodontomys* at NCC can be interpreted as reflecting unforested environments with a dense ground cover. Based on the entire fauna recorded from NCC locality, a mixed forested–grassland area surrounding permanent rivers have been inferred [23]. The specimens described here support this environmental reconstruction, and the slightly more hypsodont species of *Zygodontomys* probably mirrored unforested landscapes.

The extant sigmodontine fauna of the Urumaco region is diverse, with a dozen recognized species, mostly oryzomyine [92–97]. They can be divided between their respective habitats, from open (*Calomys hummelincki*, *Holochilus venezuelae*, *Sigmodon alstoni*, *S*. *hirsutus*, *Zygodontomys brevicauda*) to more closed areas (*Neacomys tenuipes*, *Oecomys flavicans*, *O*. *speciosus*, *O*. *trinitatis*, *Rhipidomys venezuelae*). A few are recorded from both open and forested environments (*Necromys urichi*, *Oligoryzomys delicatus*). The Pliocene evidence at NCC, with representatives of open and mixed areas, favours the hypothesis of a mixed forested–grassland area [23]. The lack of sigmodontine taxa exclusively representing closed areas might be only due to taphonomic bias, since other forms characterizing a forested environment (e.g. *Didelphis*, *Marisela*, *Proeremotherium*) have been collected [23].

### 5.3. Venezuelan fossil sigmodontines improve historical biogeography inferences

The Pliocene Venezuelan record of both *Oligoryzomys* and *Zygodontomys* poses challenging questions related to the much discussed historical biogeography of the Sigmodontinae. Whereas the more recent fossil record of this clade is well understood in its diversity and relationships with extant forms, its earlier history has been and continues to be a matter of controversy, mostly dealing with the origin and ancient history of the group [1].

It is now safe to state that sigmodontines—or their ancestors—entered South America prior to the complete formation of the Panamanian Isthmus [29,98–101]. Despite the paucity of the ancient fossil record and doubts raised about the antiquity of the materials recovered in South America [13,102–104], refined faunal analyses and absolute dates are solid, pointing to a Late Miocene age (around 5.7–6.1 Ma) for the oldest known sigmodontines [14]. Estimates of divergence times as well as the ancestral range reconstruction based on molecular clocks propose a basal split for this group much earlier, at least during the Early Middle Miocene in North or Central America [27–29,105–109]. In the most recent analysis at the genomic scale (about 3000 loci and 60 taxa), the crown age of Sigmodontinae falls at the end of the Middle Miocene (11.63 Ma [110]). There is a gap as large as 6 Myr between fossils and predictions based on genetic data and much of this ghost history is spatially associated with northern South America.

Recording living genera is crucial for exploring the evolutionary history of the sigmodontine rodents. Venezuelan fossils described here indicate that *Oligoryzomys* and *Zygodontomys* can be traced at least to the Pliocene. Only few extant genera are previously known from the Pliocene (i.e. *Akodon*, *Graomys* and *Reithrodon* [1,11]), with the communities of this epoch being mostly composed by extinct taxa (e.g. †*Cholomys*, †*Chukimys*, †*Dankomys* [1,111–113]). The above facts have several implications, one of them being the capacity of these fossils to illuminate how the mode of the evolutionary history of the sigmodontines occurred. Since genetic studies are restricted to living taxa, information about the real structure of the radiation is not solely biased to younger events but also any possibility to evaluate the impact of extinction of taxa (from genera to tribes) is mostly excluded (but see [114] for a different approach to the history of didelphid marsupials based exclusively on molecular data [115]). The Oryzomyini is the largest tribe of sigmodontine rodents that, based on morphology [5,45] and phylogenomics, is considered a basal group and the hypothesized sister clade to all other Oryzomyalia (a clade composed of at least 11 tribes plus a few incertae sedis genera [110]). The NCC record of these Oryzomyini indicates the tribe has been present in northernmost South America since the Pliocene.

One important aspect to a refreshed understanding of sigmodontine evolutionary history is to provide a better chronological control. In this sense, several clues predating the first evidence presented in this paper suggest that the entire sigmodontine radiation is younger than previously believed. This hypothesis was first advanced by Barbière *et al*. [112, p. 381], based on the oldest Argentinean fossil record. The phylogenomic analyses conducted by Parada *et al*. [110] retrieved results pointing in the same direction. At least for the origin of the Oryzomyalia, the gap between fossil record and molecular-based estimations is shortening. Faunal analyses and absolute dates [14] place the oldest oryzomyalids at about 6 Ma in central Argentina while the estimated crown age for the group is 7.36 Ma [110]. If the entire radiation of this clade, which shows a living diversity of at least 11 tribes (with the recent recognition of Rhagomyini [116]), took place in 7 Myr, several aspects must be highlighted. One aspect regarding tribal repartition is the possibility of tracing provincialism (i.e. more or less the current pattern of occupancy) to the Pliocene. *Zygodontomys* is the only genus of nonforest mammals endemic to eastern Central America and northern South America [40]. The available data are scarce but there is a rough congruence between Pliocene and Recent areas of occurrence for sigmodontine taxa.

If the contemporary radiation of the sigmodontines began during the Pliocene, the evolution of the entire subfamily (including not only oryzomyalids, but also Ichthyomyini and Sigmodontini) is rooted deeper in time [117]. The entrance of the Sigmodontinae, or its ancestors, to South America is difficult to envision without a clear understanding of palaeogeography. Several hypotheses have been proposed, such as the establishment of a connection pre-Great American Biotic Interchange or a maritime dispersal via the Caribbean Sea and the Antilles arch (see the discussion in Ronez *et al*. [1]). Although evidences are still weak to test alternative scenarios, the dispersal hypothesis through the Antilles corridor seems favoured by the Venezuelan fossil record. The northernmost portion of South America east to the Andes may have received cricetids because of its neighbouring position to the Antillean arch. More important, this part of the continent was marginal to the complex system of aquatic barriers called Pebas ([118–126] and references therein). The potential impact of the latter in the ancient history of these rodents still has not addressed in detail. However, there is a significant point that deserves to be highlighted here. Although the initial diversification of sigmodontines is viewed as a ‘burst of speciation’, an explosive and very rapid radiation [27,28,110], this picture is not totally accurate. Since the first reconstructions with moderate taxonomic coverage [105], a period of stasis was detected involving the basal portion of sigmodontine tree. During this stage, about 5 Myr long [110], just the 3 main branches composing the living expression of the subfamily (i.e. Ichthyomyini, Oryzomyalia and Sigmodontini [116]) are present. Then, around 7–6 Ma, the ‘explosive’ radiation of the oryzomyalids occurred. The most parsimonious way to explain this two-step structure is to suppose that the initial wave of colonization was ‘contained’ by a main geographical barrier. The Pebas system, almost isolating northern South America ([127] and references therein), could have played the role of such a barrier.

After the initial establishment of the sigmodontines on the continent, several drivers have been proposed as important in promoting the diversification of these rodents [128]. One of the first models to be advanced on this issue was that of Marshall [25] where open areas (savannahs) developed to the east of the Andes and played a crucial role aiding or obstructing Late Miocene–Pliocene sigmodontine dispersion southwards. According to this hypothesis, savannah/grassland landscapes allowed the interchange between north and central/south portions of the continent favouring the progressive dispersion of pastoral cricetids (*sensu* Hershkovitz [5]). The extensive territories exposed after the regression of the Paranean Sea, during the still poorly known ‘Age of the Southern Plains’ [129], perhaps acted as a ‘pump of speciation’ of sigmodontines. At least in central Argentina, the rising of C4 plants is framed within this time-window (11 to 3 Ma), characterized by a gradual decrease in temperature and the spread of grasslands [130]. Marshall's model does not conflict with an arrival from the Antilles and a first stage of isolation on the Guyana shield and northern neighbouring areas. Nevertheless, even when our understanding of Andean and Amazonian Neogene palaeogeography dramatically increased during the last four decades ([127] and references therein), there have been few additions to our knowledge of the sigmodontine fossil record. One promising finding is the sigmodontine †*Cordimus*, a genus with three species described from Quaternary deposits in the Caribbean islands of Curaçao & Bonaire [131]. Although one of the species, †*Cordimus hooijeri*, restricted to the Late Holocene in Bonaire seems unrelated to the other two (resembling more probably *Oligoryzomys*), at least the type species of the genus deserves closer attention. It is †*Cordimus debuisonjei*, collected in Curaçao, from sediments supposedly bracketed between 2.3 and 1.3 Ma [131]. This form, characterized by brachydont complex molars resembling oryzomyines in occlusal morphology, shows several unusual traits among sigmodontines, including the single condition (i.e. being constituted by only one conulid [38]) on the procingulum of the m1 associated with the absence of mesolophid on this same molar (fig. 2*d* in [131]). The phylogenetic affinities of †*Cordimus* are unknown as well as its tribal affiliation, although it was compared to †*Copemys* and several North American fossil Miocene muroids [131]. Maybe †*Cordimus* could be an important piece of information to support a Caribbean dispersion to South America. Several attempts to perform a direct study of these important fossils failed. Palaeontological collections in the Naturalis Biodiversity Center in Leiden (The Netherlands), where †*Cordimus* was housed, were in relocation (N. Den Ouden 2018, 2019 and 2020, personal communication to U.F.J.P.), and then the materials were lost (L. Van Den Hoek Ostende 2020 and 2021, personal communication to U.F.J.P.).

The current distribution of the 13 tribes recognized for the Sigmodontinae clearly points to a complex history where forested or unforested areas, mountain chains, and main river systems played a major role [3]. Although hypothesized ancestral range reconstructions for the ancestors of the main tribes of sigmodontines, from those originally proposed by Reig [6,132] to recent estimations based on molecular data [108] retrieve areas spatially linked to the Andes, there is a growing perception that eastern South America was crucial in the evolution of these rodents. A refined scenario is emerging regarding both Caribbean tectonics [133–135] and Amazonian Neogene evolution [136,137], and this constitutes an opportunity to put in context and rework previous hypotheses regarding historical biogeography. Although treated as Oryzomyini after the influential revision made by Voss [40], *Zygodontomys* was traditionally viewed as a form with controversial phylogenetic affinities [5]. We hypothesize that *Zygodontomys* is the single survivor of an unrecognized tribe with ancient roots in northern South America [49]. This enhances the results of Weksler [41, p. 75]: ‘the recovery of this clade and its position at a basal branch within oryzomyines were unexpected. *Scolomys* and *Zygodontomys* are two of the most distinctive clades of oryzomyines, and they are ecologically and morphologically dissimilar from one another’. It has also been concluded by Percequillo *et al*. [138] that both genera may have differentiated from Oryzomyini during the Late Miocene. Finally, the Venezuelan Pliocene occurrence of *Zygodontomys* gives strong temporal support to a unique unsuspected radiation among sigmodontines [139].

## 6. Conclusion

The Late Pliocene Norte Casa Chiguaje locality yielded many fossils representing a diverse assemblage including sigmodontine rodents, the most speciose subfamily of Neotropical mammals. Two oryzomyine genera, *Oligoryzomys* sp. and *Zygodontomys* sp., are recognized from the locality, which represent the first Pliocene record of sigmodontines outside Argentina. From a palaeoenvironmental point of view, the presence of both genera reinforces the occurrence of mixed forested–grassland areas advanced by the entire fauna as described by Carrillo-Briceño *et al*. [23]. Biogeographically, the occurrence of fossil sigmodontines in northern Venezuela supports the possibility of a dispersal of their ancestors through the Antilles corridor, as well as the importance of open areas/savannahs to their evolution. More fossils are needed from this portion of the continent to reinforce or correct the hypotheses about the time and diversification of the sigmodontine rodents which are today almost exclusively based on molecular phylogenies [108–110,138,140]. The positive results obtained by screen-washing sediments from NCC are eloquent and indicate there is much more to be learned from this neglected record.

## Data Availability

The datasets supporting this article (electronic supplementary material, S1, figures S1–S3) have been uploaded and are available from the Dryad Digital Repository: https://doi.org/10.5061/dryad.prr4xgxqb [141]. The data are provided in electronic supplementary material [142].
